# *In Situ* Effects of 4,6-α- and 4,3-α-Glucanotransferases During Sourdough Fermentation: Assessing Microbial Community Dynamics, Bread Glycemic Index, Staling, and Texture

**DOI:** 10.3390/foods15152757

**Published:** 2026-08-05

**Authors:** Duygu Zehir-Şentürk, Furkan Demirgül, Ramazan Tolga Niçin, Redife Aslıhan Ucar, Ömer Şimşek

**Affiliations:** 1 Department of Food Engineering, Faculty of Engineering, Pamukkale University, Denizli 20160, Türkiye; dzehir3455@gmail.com; 2 Department of Gastronomy and Culinary Arts, Faculty of Arts and Design, Dogus University, Istanbul 34775, Türkiye; fdemirgul@dogus.edu.tr; 3 Department of Food Engineering, Chemical and Metallurgical Faculty, Yıldız Technical University, Davutpaşa Campus, Istanbul 34210, Türkiye; nicin.ramazan@gmail.com (R.T.N.); redifeaslihan.ucar@gmail.com (R.A.U.); 4 Food Control Laboratory Directorate, Republic of Türkiye Ministry of Agriculture and Forestry, Denizli 20010, Türkiye; 5 Denizli Governorship, Denizli 20059, Türkiye

**Keywords:** sourdough, lactic acid bacteria, α-glucanotransferase, starch retrogradation, glycemic index, textural properties, food safety

## Abstract

Although some lactic acid bacteria species harbor genes encoding α-glucanotransferases (4,6-α-GTase and 4,3-α-GTase), the technological and functional implications of these enzymes within sourdough ecosystems remain poorly understood. This study investigated the effects associated with α-GTase-positive and α-GTase-negative strains of *Limosilactobacillus reuteri* and *L. fermentum* on sourdough microbiota, bread quality, and starch digestibility. Starter culture inoculation steered the assembly of the sourdough microbiota, resulting in stable acidification and altered microbial community structures. High-throughput sequencing analysis showed that starter culture addition generally increased taxonomic richness, while Shannon and Simpson diversity indices varied according to strain genotype and inoculation level. Sourdoughs fermented with α-GTase-positive strains exhibited higher viscosity while maintaining shear-thinning behavior, which was putatively linked to *in situ* enzymatic synthesis. Additionally, inoculation with these strains improved bread specific volume, suggesting a possible structural modification of the starch matrix or the overlapping effects of fermentation metabolites. During storage, the 4,3-α-GTase-positive *L. fermentum* PFC282 strain delayed the increase in bread hardness, reflecting a possible mitigation of amylopectin retrogradation and indicating a potential anti-staling effect. In addition, the 4,6-α-GTase-positive *L. reuteri* PFC338 strain produced breads with lower estimated glycemic index values, suggesting a lower susceptibility to enzymatic starch hydrolysis likely due to the hypothesized alterations in structural linkages. Overall, the findings highlight the potential of α-GTase-positive sourdough starter cultures to modulate the technological and nutritional properties of bread.

## 1. Introduction

Sourdough fermentation is a traditional biotechnological process driven by the symbiotic activity of lactic acid bacteria (LAB) and yeasts, which improves nutrient bioavailability and shelf life of cereal-based products [1]. Although starch, the predominant component of the complex sourdough matrix, provides essential technological functionalities in bakery products, its inherent structural limitations, such as low solubility in dough systems and high susceptibility to retrogradation, can often compromise end-product quality [2]. Driven primarily by the recrystallization of amylopectin chains, starch retrogradation is a fundamental cause of bread staling, leading to substantial economic losses and food waste in the baking industry [3]. Furthermore, while sourdough fermentation can lower the estimated glycemic index (eGI) of bread, the high-glycemic nature of starch-rich bakery formulations remains a persistent challenge [4]. Consequently, targeted structural modification of starch is imperative to impart desirable physicochemical and functional attributes, thereby optimizing its industrial compatibility [5,6].

Structural drawbacks of native starch can be mitigated by utilizing chemical, physical, or biotechnological (specifically enzymatic) modifications, either individually or in combination [7,8,9]. Among these, enzymatic approaches have attracted considerable interest, driven by the growing consumer demand for clean-label products and the need to minimize chemical waste or hazardous by-products [10]. In this context, enzymes of the glycoside hydrolase family 70 (GH70) have gained increasing interest as natural tools for tailoring the structural and functional properties of starch. Traditionally known for glucansucrases that synthesize α-glucans from sucrose, this family also encompasses specialized α-glucanotransferases (α-GTases) [11]. Produced by specific LAB strains, these enzymes (4,6-α-GTase and 4,3-α-GTase) possess the unique capability to modify starch architecture by converting linear α-1,4-glucosidic linkages into branched α-1,6 or α-1,3 linkages, respectively [12]. This enzymatic rearrangement introduces novel branching points that exert steric hindrance against the realignment of amylopectin chains, thereby suppressing retrogradation enthalpy and delaying staling [13]. Furthermore, the resulting complex branched matrices and malto-oligosaccharides exhibit enhanced resistance toward human digestive enzymes, consequently lowering the glycemic index [14,15].

In our previous study [16], genes encoding 4,6-α-GTase and 4,3-α-GTase from *Limosilactobacillus reuteri* E81 (reclassified as *L. reuteri* PFC338) and *L. fermentum* PFC282, respectively, were successfully expressed in *Lactococcus lactis* MG1363 to investigate their glycosidic bond-forming capabilities and catalytic efficiencies on starch substrates. In a subsequent study exploring the direct application of these purified enzymes as additives in bakery products [17], they were found to alter starch granule morphology, delay staling without compromising sensory quality, and reduce the eGI values of bread by 18.01% (4,6-α-GTase) and 13.61% (4,3-α-GTase). However, the industrial-scale utilization of purified or partially purified enzymes is often limited by substantial production costs and the requirement for food additive declarations. Consequently, shifting from direct enzyme supplementation to an *in situ* production strategy within the sourdough ecosystem offers a highly sustainable, cost-effective, and clean-label alternative.

Although certain LAB strains are documented as α-GTase producers in the literature [12], to the best of our knowledge, no study has yet investigated the *in situ* production of these specific enzymes within a sourdough matrix. To address this gap, the present study aimed to utilize *L. reuteri* PFC338 and *L. fermentum* PFC282, natural producers of 4,6-α-GTase and 4,3-α-GTase, respectively, as sourdough starter cultures, and to comprehensively evaluate their impacts on the microbiota, eGI, and staling properties of both sourdough and the resulting bread. This novel approach seeks to provide insights into the potential role of *in situ* enzymatic modification on the sourdough matrix and its subsequent macromolecular outcomes.

## 2. Materials and Methods

### 2.1. Screening and Cultivation of Starter Strains Based on α-Glucanotransferase Genes

The LAB strains utilized in this study were selected from the Pamukkale University Food Engineering Culture Collection (PUFECC) based on their potential to produce α-GTase. The presence of genes encoding 4,6-α-GTase (*gtfB1*) and 4,3-α-GTase (*gtfB2*), which were characterized in our previous study [16], was confirmed in candidate strains by colony PCR. Species-specific primer pairs used for the PCR assays were as follows: GTFB1-M-ECORI-F1 (5′-ACA AGC CAG ATG GTA AGA C-3′) and GTFB2-M-KPNI-R1 (5′-CGG TTA ACA ATA CTA-3′) for *L. reuteri* strains; GTFB1-F (5′-ACA AGC CCA RGA TGG TAA-3′) and GTFB2-R (5′-TCG CCA TTA GST AAT TT-3′) for *L. fermentum* strains. Amplification reactions were performed in a total volume of 50 µL containing 10 µM of each primer, 10 mM dNTP mix (Merck, Darmstadt, Germany), and OneTaq DNA Polymerase (New England Biolabs, Ipswich, MA, USA), following a protocol adapted from the manufacturer’s instructions. The thermal cycling profile consisted of an initial denaturation at 94 °C for 1.5 min, followed by 30 cycles of denaturation at 95 °C for 30 s, annealing at the specific optimized melting temperature (Tm −5 °C) of each primer pair for 1 min, and extension at 68 °C for 60 s/kb, with a final extension at 68 °C for 5 min. PCR products were resolved by 1% (*w*/*v*) agarose gel electrophoresis (Merck, Germany). Strains yielding specific bands of the expected molecular size were designated as α-GTase-positive, while those without bands were classified as α-GTase-negative. Consequently, 4,6-α-GTase-positive *L. reuteri* PFC338, α-GTase-negative *L. reuteri* PSC73, 4,3-α-GTase-positive *L. fermentum* PFC282, and α-GTase-negative *L. fermentum* PFC268 were selected for further use. The α-GTase-negative strains were utilized as strain-specific baseline controls due to their comparable sourdough acidification profiles and microbial growth kinetics relative to the α-GTase-positive strains, thereby aiming to minimize fermentative variations. Stock cultures were maintained at −80 °C in MRS broth (Merck, Germany) supplemented with 20% (*v*/*v*) glycerol (Riedel-de Haën, Seelze, Germany).

### 2.2. Preparation of Sourdoughs

To prepare the sourdough samples, the α-GTase-positive and α-GTase-negative starter cultures were inoculated into MRS broth and incubated until the late-exponential phase at 30 °C for *L. fermentum* strains and at 37 °C for *L. reuteri* strains. Bacterial cells were harvested by centrifugation (Hitachi Centrifuge, Tokyo, Japan) at 9000 rpm for 30 min, washed twice with sterile PBS buffer, and adjusted to an OD_600nm_ of 1.0. The cell suspensions were then serially diluted in sterile PBS to reach the target initial cell counts. Sourdoughs were prepared with a dough yield of 200 (*w*/*w*) using commercial additive-free white wheat flour (Carrefoursa, Istanbul, Türkiye) and potable water. According to the manufacturer’s specifications, the flour contained 10.4% protein, 71.9% carbohydrate (2.9% sugars), 3.5% dietary fiber, and 1.1% fat, with a maximum ash content of 0.7% (dry basis). In terms of functional properties, it exhibited a water absorption capacity of 56.23% as determined by Farinograph analysis. Initially, a master dough was prepared and divided into equal portions. According to the experimental design (Table 1), each dough portion was inoculated with 4,6-α-GTase-positive, 4,6-α-GTase-negative, 4,3-α-GTase-positive, or 4,3-α-GTase-negative strains at two different initial inoculation levels (10^2^ and 10^6^ CFU/g). Sourdough prepared without any starter culture inoculation served as the spontaneously fermented control group. Fermentation was carried out at 30 °C until a target pH of 4.5 ± 0.1 was achieved.

Upon reaching the target pH, a 10% (*w*/*w*) aliquot of the fermented sourdough was propagated into a fresh flour–water mixture, and this backslopping process was repeated for three consecutive cycles. At the end of each backslopping stage, fermented sourdough samples were collected aseptically and stored in sterile falcon tubes at −20 °C and protected from light and moisture, specifically for subsequent microbiological counting and DNA extraction.

### 2.3. Total Titratable Acidity

The total titratable acidity (TTA) values of the sourdough samples were determined at each backslopping cycle (BC). To measure TTA, a 10 g dough sample was homogenized in 100 mL of distilled water. The suspension was then titrated with 0.1 N NaOH (Merck, Germany) using a 1% phenolphthalein indicator (Merck, Germany) under continuous stirring until a faint pink color persisted for 30 s. The total acidity of the samples was calculated from the volume of titrant consumed and expressed as a percentage of lactic acid equivalent (%).

### 2.4. Microbiological Analysis

At each BC, the fermented sourdough samples were homogenized with sterile peptone physiological saline at a 1:10 (*w*/*v*) ratio using a stomacher blender (Seward Medical, London, UK), and subsequent ten-fold serial dilutions were prepared. LAB were enumerated on MRS agar supplemented with 0.01% cycloheximide and GM17 agar (containing 0.5% glucose, Condalab, Madrid, Spain) following incubation at 30 °C for 72 h. Total aerobic mesophilic bacteria (TAMB) counts were determined on Plate Count Agar (PCA; Merck, Germany) after incubation at 30 °C for 72 h. Yeasts were enumerated on Dichloran Rose Bengal Chlortetracycline (DRBC) agar (Merck, Germany) after an incubation period of 3–5 days at 25 °C. All microbiological counts were expressed as log CFU/g of sourdough.

### 2.5. Culture-Independent PCR-DGGE Profiling of LAB in Sourdoughs

To profile the LAB diversity within the sourdough samples, a culture-independent denaturing gradient gel electrophoresis (DGGE) approach was employed, modified from the protocols described by Van der Meulen et al. [18] and Oguntoyinbo and Dodd [19]. For microbial genomic DNA extraction, sourdough samples were homogenized with peptone water at a 1:10 (*w*/*v*) ratio. To separate large dough particles from the microbial fraction, the homogenate was initially centrifuged at 1000× *g* for 5 min. The resulting supernatant was then subjected to a second centrifugation at 5000× *g* for 15 min to harvest the bacterial cells. The recovered cell pellets were washed twice with PBS buffer, and genomic DNA was isolated using the Bacterial & Yeast Genomic DNA Purification Kit (EurX, Gdańsk, Poland). The V3 region of the bacterial 16S rDNA was amplified using the GC-clamped forward primer P338F and the reverse primer P518R. PCR amplifications were conducted in a total reaction volume of 40 µL, containing 8 µL of 5X FIREPol^®^ Master Mix (Solis BioDyne, Tartu, Estonia), 1 µM of each primer, and 2 µL of template DNA. The thermal cycling profile consisted of an initial denaturation at 95 °C for 12 min, followed by 30 cycles of denaturation at 95 °C for 20 s, annealing at 55 °C for 45 s, and extension at 72 °C for 1 min, with a final extension at 72 °C for 5 min. DGGE analysis was performed on a vertical electrophoresis system (Thermo Fisher Scientific, Waltham, MA, USA) using polyacrylamide gels formulated with a 30% to 60% denaturant gradient (where 100% denaturant corresponds to 7 M urea and 40% formamide). Electrophoresis was carried out in 1X TAE buffer at a constant temperature of 60 °C, initially at 50 V for 15 min, followed by a separation phase at 150 V for 4 h. Finally, the gels were stained with SYBR Gold (Invitrogen, Carlsbad, CA, USA) and visualized under UV illumination.

### 2.6. High-Throughput 16S rRNA Amplicon Sequencing of Sourdough Microbiota

At the end of BC_3_ (pH 4.5 ± 0.1), total genomic DNA was extracted from three independent biological sourdough replicates and evaluated according to standard quality criteria regarding concentration and purity. To accommodate sequencing optimization and represent the baseline microbial profile of the master fermentations while managing technical variations, equal amounts of purified DNA from the biological replicates were pooled into a single representative composite sample per group prior to sequencing. To elucidate bacterial diversity, high-throughput sequencing of the V3 region of the 16S rRNA gene was performed. The V3 region was amplified using specific primers and purified. During library preparation, Illumina dual indices and sequencing adapters were appended using the Nextera XT Index Kit (Illumina, San Diego, CA, USA), followed by a secondary purification step. The concentration of the constructed libraries was quantified via real-time PCR (qPCR), and all samples were normalized by dilution to a final concentration of 4 nM. Normalized libraries were subsequently pooled. Amplicon sequencing was performed on the Illumina platform using a 2 × 250 bp paired-end read length based on the sequencing-by-synthesis principle. The resulting fluorescence data were converted into raw FASTQ files. All bioinformatics analyses were executed using the QIIME 2 (v2018.11) platform [20]. As part of the data quality control pipeline, reads with a Phred score below 20, along with primers and barcodes, were trimmed and filtered. After removing chimeric sequences, Amplicon Sequence Variants (ASVs) were resolved using the DADA2 plugin within QIIME 2. Taxonomic classification of the filtered sequences was performed against the SILVA database. Sequences matching plant organelles (chloroplasts and mitochondria) originating from the wheat flour matrix were filtered out prior to downstream analyses. Alpha and beta diversity analyses were carried out utilizing the QIIME 2 core-diversity pipelines to determine intra- and inter-sample diversity. Rarefaction curves were constructed to verify whether the sequencing depth sufficiently represented the microbial diversity within the sourdough matrix. The curves generally appeared to approach a plateau at the selected standardized sequencing depth threshold of 2000 reads per sample, suggesting that the sub-sampling depth was adequate to capture the core microbial community structure (Figure S1). Due to the pooling of biological replicates into a single representative composite sample per group, alpha and beta diversity values are reported descriptively to present the baseline microbial profile of each formulation without inferential group comparisons.

### 2.7. Breadmaking from Sourdoughs

Bread production was carried out in three independent batches for each formulation using sourdoughs prepared according to the experimental design (Table 1) that reached the target pH (4.5 ± 0.1) at the end of BC_3_. The bread dough formulation consisted of commercial additive-free white wheat flour (Carrefoursa, Türkiye) on a 14% moisture basis, 1.5% table salt, and 2% commercial fresh yeast (Pakmaya, Istanbul, Türkiye) calculated on a flour basis. These ingredients were initially blended at a low speed (speed setting 2) for 1 min using a KitchenAid mixer (KitchenAid, Greenville, OH, USA), after which matured sourdough was incorporated into the matrix at a level of 15% (*w*/*w*). The specific water addition was determined by reducing the water absorption capacity obtained from Farinograph analysis by 2%. Upon adding the calculated amount of water based on flour weight, the dough was kneaded for 5 min. The resulting dough was subjected to bulk fermentation at room temperature (25 °C ± 1.0) for 30 min at a relative humidity above 80%. Following the bulk fermentation, the dough from each independent batch was divided into 160 g pieces, allowed to rest for 10 min, shaped, and placed into baking molds. The final proofing stage was conducted at 30 °C for 45 min, and the loaves were subsequently baked at 220 °C for 17 min. The produced breads were stored in polyethylene bags within a climate cabinet (Nüve-TK-120, Ankara, Türkiye) maintained at 25 °C and 65% relative humidity. For the accelerated staling and thermal properties analyses, a separate set of bread samples was kept under controlled storage at 4 °C.

### 2.8. Viscosity and Rheological Properties of Sourdoughs

The apparent viscosity of sourdough samples (at day 0 and mature preferment stage) was measured using a rotational viscometer (Brookfield Model RVDV-II, Middleboro, MA, USA) equipped with an SC4-14 spindle at 25 °C. The measurements were performed over a progressive rotational speed range (1 to 100 rpm) to obtain the shear stress and shear rate data. To describe the steady-shear flow behavior of the sourdoughs, the experimental data were fitted to the Power Law (Ostwald–de Waele) model:

*τ* = *K* (*γ*)*^n^*

where *τ* is the shear stress (Pa), *K* is the consistency index (Pa.s*^n^*), *γ* is the shear rate (s^−1^), and *n* is the flow behavior index.

### 2.9. Specific Volume of Sourdough Breads

The specific volumes of the breads were determined using a rapeseed displacement method modified from Kazakos et al. [21]. The values were calculated by dividing the loaf volume by its weight.

### 2.10. Textural Properties of Sourdough Breads

Texture profile analysis (TPA) of the breads was performed during storage (days 0, 3, 5, and 7) using a texture analyzer (Brookfield CT3-4500, Middleboro, MA, USA) equipped with a TA3/1000 cylindrical probe, following a modified method of Sun et al. [22]. Slices of 18 mm thickness were cut from the center of the loaves. The samples were subjected to a two-cycle compression test at 50% deformation with a trigger force of 0.07 N and a test speed of 1 mm/s. Hardness, chewiness, cohesiveness, gumminess, and springiness values were calculated from the resulting force–time curves.

### 2.11. Thermal Properties of Sourdough Breads

The thermal properties of retrograded amylopectin in the bread samples were determined using a differential scanning calorimeter (DSC131 EVO, Setaram, Caluire-et-Cuire, France) following a modified method of Hadaegh et al. [23]. Approximately 3.2 mg of bread crumb sample (stored at 4 °C at days 0, 3, 5, and 7) was weighed directly into aluminum pans, hermetically sealed, and equilibrated prior to analysis. The measurements were carried out under an argon atmosphere from 25 °C to 135 °C at a constant heating rate of 5 °C/min.

### 2.12. Estimated Glycemic Index of Sourdough Breads

The estimated glycemic index (eGI) of the bread samples was determined via an *in vitro* starch hydrolysis procedure based on the methods described by Englyst et al. [24] and Yaman et al. [25]. Briefly, 1 g of homogenized bread sample was mixed with 5 mL of distilled water and 10 mL of a pepsin (Sigma-Aldrich, St. Louis, MO, USA)–guar gum (Merck, Germany) solution, and incubated at 37 °C and 175 rpm for 30 min. Following the gastric phase, the pH was adjusted to 5.0–5.25 using a 0.5 M sodium acetate solution (Merck, Germany). An enzyme mixture containing pancreatin (Sigma-Aldrich, USA) and amyloglucosidase (13.4 U/mL; Megazyme, Bray, Ireland) was then added, and the total volume was completed to 50 mL. During the 180 min intestinal incubation, 0.5 mL aliquots were collected at specific time intervals (20, 30, 60, 90, 120, and 180 min) and immediately immersed in a boiling water bath for 5 min to terminate enzyme activity. After centrifugation (4000× *g*, 5 min), the glucose content in the supernatant was measured spectrophotometrically (UV-1800, Shimadzu, Tokyo, Japan) at 510 nm using a glucose determination kit (GOPOD-format, Megazyme, Ireland). To account for differences in moisture among the samples, all calculations were corrected based on the dry matter content of the breads (on an equal dry weight basis). Commercial white wheat bread crumb (crust-free) was utilized as the reference bread. The area under the hydrolysis curve (AUC) for both the samples and the reference bread was calculated using the trapezoidal rule. The hydrolysis index (HI) was calculated using the following formula:

$$\mathrm{HI}=\left(\frac{\mathrm{AUC}\;\mathrm{of}\;\mathrm{the}\;\mathrm{sample}}{\mathrm{AUC}\;\mathrm{of}\;\mathrm{the}\;\mathrm{reference}\;\mathrm{white}\;\mathrm{bread}}\right)\times 100$$

Consequently, the HI value of the reference white bread was defined and set as 100.00. The eGI values were obtained using the equation proposed by Goñi et al. [26] and Yaman et al. [25]:

eGI = 39.71 + (0.549 × HI)

These estimated values, representing predicted glycemic responses based on in vitro starch hydrolysis, were classified into three categories according to the adapted FAO/WHO international guidelines: low-glycemic (<55), medium-glycemic (55–69), and high-glycemic (≥70) categories [27].

### 2.13. Statistical Analysis

Sourdough fermentation and bread baking processes were executed in three independent biological batches. Analytical measurements were performed in technical triplicates for each individual batch. The technical replicates within each biological batch were averaged, and the resulting mean values from the three independent batches were utilized as the replicates (n = 3). The data were expressed as mean ± standard deviation (SD). Prior to ANOVA, the assumptions of normality and homogeneity of variance were verified using the Shapiro–Wilk test and Levene’s test, respectively. To evaluate the differences among the samples, a two-way analysis of variance (ANOVA) was performed for storage-dependent parameters, while one-way ANOVA was applied for fermentation monitoring parameters and the remaining physicochemical and functional properties. For post hoc multiple comparisons, the Bonferroni correction was applied following the two-way ANOVA, whereas Tukey’s test was employed after the one-way ANOVA to determine significant differences between the groups. All statistical analyses were conducted using IBM SPSS Statistics software (Version 26, Armonk, NY, USA), and the level of statistical significance was set at *p* < 0.05. High-throughput 16S rRNA amplicon sequencing data were excluded from these inferential statistical analyses due to sample pooling and were reported descriptively.

## 3. Results

### 3.1. Genotypic Verification of α-GTase Genes in Starter Strains

PCR analysis targeting the 4,6-α-GTase (*gtfB1*) and 4,3-α-GTase (*gtfB2*) genes within the candidate genomes confirmed the presence of these genetic determinants, clearly showing that specific amplification fragments of approximately 1000 bp successfully accumulated for *L. reuteri* PFC338 (4,6-α-GTase-positive) and *L. fermentum* PFC282 (4,3-α-GTase-positive). Conversely, no amplification product was present in the genomes of *L. reuteri* PSC73 (4,6-α-GTase-negative) and *L. fermentum* PFC268 (4,3-α-GTase-negative). These results genotypically verified the potential of the positive strains to encode for α-GTase activity, establishing them as suitable models for evaluating in situ technological effects.

### 3.2. Monitoring of Sourdough Fermentation

Table 2 summarizes the TTA values of the sourdoughs fermented at 30 °C upon reaching the target pH of 4.5.

TTA values revealed that starter culture formulations, inoculation dosages (10^2^ and 10^6^ CFU/g), and consecutive BCs exerted a statistically significant effect (*p* < 0.05) on acid accumulation (Table 2). Prior to fermentation (T_0_), the dough samples generally exhibited similar (*p* > 0.05) TTA levels (0.58–0.70% expressed as lactic acid), whereas only SD_282H_ (0.40%) showed a significantly lower (*p* < 0.05) initial acidity. Throughout all BCs (BC_1_, BC_2_, and BC_3_), SD_338H_ stood out with the highest TTA values (0.71–0.72%).

Starter culture formulations and inoculation dosages significantly influenced (*p* < 0.05) all microbial counts (LAB on MRS and GM17, TAMB, and yeast–mold) across all BCs (Table 2). Prior to fermentation (T_0_), LAB counts ranged between 8.00 and 9.15 log CFU/g on MRS and 8.10 and 9.20 log CFU/g on GM17. By BC_3_, the LAB counts maintained stability (8.64–9.30 log CFU/g on MRS; 8.53–9.30 log CFU/g on GM17), where samples SD_338H_ and SD_268L_ secured the highest cell loads. Similarly, TAMB remained stable throughout consecutive passages, shifting from an initial 8.00–9.30 log CFU/g (T_0_) to 8.38–9.15 log CFU/g at BC_3_. In contrast, yeast–mold counts declined sharply; while sample SD_282L_ had the highest initial load (4.54 log CFU/g) and two samples (SD_268H_ and SD_282H_) showed no initial growth at T_0_, the yeast–mold counts dropped below the detection limit in six out of nine samples by the final cycle (BC_3_).

### 3.3. DGGE Characterization of the Microbial Profile

**Figure 1:**
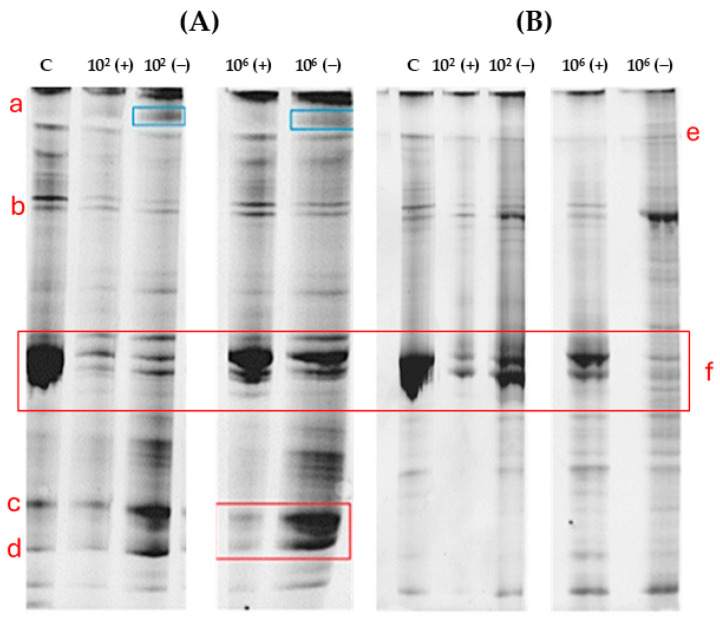
DGGE gel profiles of LAB in sourdoughs at the end of the 3rd backslopping cycle at 30 °C. (**A**) Sourdoughs fermented with 4,6-α-GTase-positive and 4,6-α-GTase-negative variants, (**B**) sourdoughs fermented with 4,3-α-GTase-positive and 4,3-α-GTase-negative variants. Lanes: C: Spontaneous control; 10^2^ (+): 10^2^ CFU/g active variant; 10^2^ (−): 10^2^ CFU/g inactive variant; 10^6^ (+): 10^6^ CFU/g active variant; 10^6^ (−): 10^6^ CFU/g inactive variant. The lanes corresponding to the 10^4^ CFU/g inoculation dose were excluded from the layout per the final experimental design. Lowercase letters (a–f) and the blue and red frames indicate representative DGGE bands or band regions.

DGGE gel analysis revealed shifts in LAB profiles of the sourdough samples fermented with 4,6- and 4,3-α-GTase variants at different inoculation dosages at the end of BC_3_ (Figure 1). Although each well received an equal concentration (20 ng) of PCR product (DNA), the resulting specific band profiles and intensities displayed variations depending on the starter cultures and inoculation dosages.

DGGE band profiles of sourdoughs fermented with *L. reuteri* strains showed intensity variations (Figure 1A). Band “a”, which remained weak in the SD_C_, SD_338L_, and SD_338H_ samples, displayed higher relative intensity in SD_73L_ and SD_73H_. Meanwhile, band “b”, prominent in SD_C_, weakened in both low-dose samples (SD_73L_ and SD_338L_); however, it intensified in SD_338H_ but was less intense in SD_73H_. In region “f”, the starter inoculation led to a reduction in the intensity of the thick band cluster present in SD_C_. For the common bands “c” and “d”, the α-GTase-negative strains (SD_73L_ and SD_73H_) had wider and darker profiles than their positive counterparts (SD_338L_ and SD_338H_), reaching maximum width and peak intensity in SD_73H_ (Figure 1A). For sourdoughs fermented with *L. fermentum* strains (Figure 1B), band “b” showed higher intensity in SD_268L_ and SD_268H_ compared to both SD_C_ and the SD_282_ counterparts. Similar to Figure 1A, *L. fermentum* strains inoculation decreased the intensity of the band structure in region “f” across all lanes relative to SD_C_. Conversely, bands “c” and “d”, which were strong in *L. reuteri* strain profile, were nearly undetectable or fainter in all *L. fermentum* samples, reflecting potential species-specific variations. Additionally, band “e”, prominent especially in high-dose *L. reuteri* samples (SD_338H_ and SD_73H_), remained faint in all *L. fermentum* treatments.

### 3.4. 16S rRNA Gene Sequencing Data and Quality Metrics

High-throughput 16S rRNA gene sequencing yielded a total of 1,252,423 high-quality reads to resolve the microbial composition and diversity of the sourdough samples. The filtered read count per sample varied between 99,409 and 179,553, with an average of 139,158 reads. The proportion of high-quality reads retained after the bioinformatic filtering pipeline suggested satisfactory library quality and provided sufficient sequencing depth for downstream taxonomic characterization.

### 3.5. Bacterial Community Stability and Alpha Diversity Analysis

**Figure 2:**
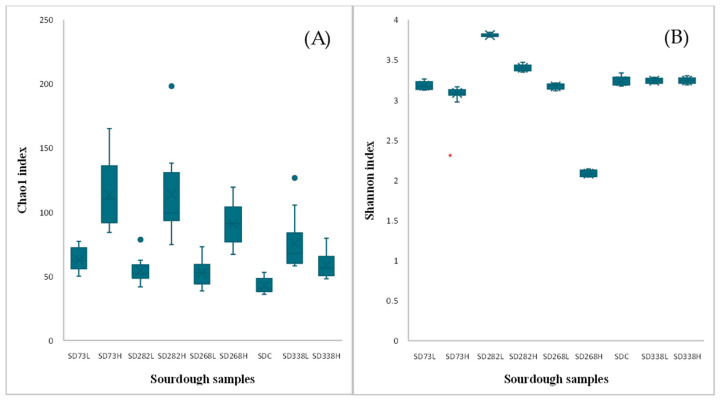
Alpha diversity indices of sourdough bacterial communities across different groups. (**A**) Chao1 index representing species richness, and (**B**) Shannon index representing bacterial diversity. Box plots indicate the median, quartiles, and range of 10 computational rarefaction iterations performed at a standardized sequencing depth of 2000 reads per sample. Outliers are represented by individual points. SD_338_: *L. reuteri* PFC338 (4,6-α-GTase-positive); SD_73_: *L. reuteri* PSC73 (4,6-α-GTase-negative); SD_282_: *L. fermentum* PFC282 (4,3-α-GTase-positive); SD_268_: *L. fermentum* PFC268 (4,3-α-GTase-negative). Subscripts “L” and “H” represent low (10^2^ CFU/g) and high (10^6^ CFU/g) initial inoculation levels, respectively.

Table 3 and Figure 2 show the bacterial richness and diversity indices (Chao1, Shannon, and Simpson) of the composite pooled sourdough samples. Good’s coverage values were at or above 98.48% for all samples, suggesting that the standardized rarefaction subset of 2000 reads per sample was adequate to represent the primary taxonomic profiles. Aligned with these metrics, the rarefaction curves approached a plateau, indicating that the sequencing depth captured the bacterial diversity within the groups.

The bacterial richness (Chao1) of the sourdough samples varied among the starter culture formulations and inoculation dosages. The spontaneous control group (SD_C_) exhibited the lowest taxonomic richness with a value of 42.62, while starter culture incorporation generally increased the taxonomic richness of the sourdoughs (Figure 2A). In line with these variations, the Shannon and Simpson indices, which reflect the diversity and dominance profiles, showed shifts based on strain variants. SD_282L_ exhibited the highest Shannon index (3.81) along with a Simpson dominance value of 0.2575, suggesting potential changes in taxonomic distribution within the ecosystem. Conversely, SD_268H_ displayed the lowest diversity metrics, with a Shannon index of 2.08 and a Simpson value of 0.0584 (Figure 2B). Specifically, the enzyme-active strain at a low dosage (SD_282L_) contributed to a different profile, establishing a high Shannon index despite a higher microbial dominance profile, particularly when comparing the 4,3-α-GTase variants, whereas the 4,3-α-GTase-negative strain at a high inoculation level (SD_268H_) influenced the overall uniformity and dominant profiles in the sourdough ecosystem.

### 3.6. Bacterial Community Composition and Taxonomic Distribution

**Figure 3:**
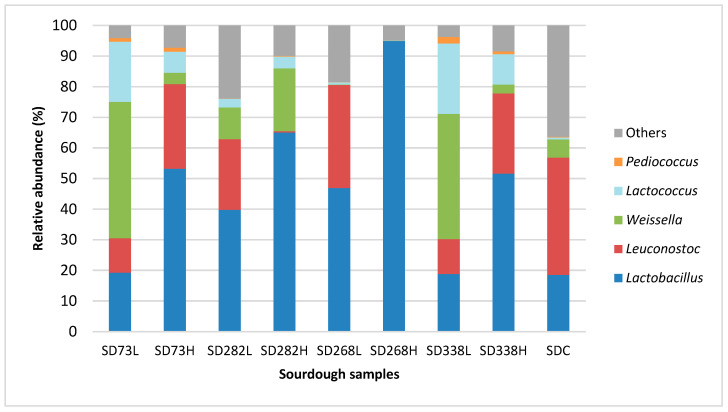
Taxonomic composition of the bacterial communities at the genus level across different sourdough groups, presenting the relative abundance (%) of primary bacterial genera. Sequences originating from plant organelles (mitochondria and chloroplasts) were filtered out prior to analysis, and genera with an individual abundance below 0.5% were pooled into the “others” group. SD_338_: *L. reuteri* PFC338 (4,6-α-GTase-positive); SD_73_: *L. reuteri* PSC73 (4,6-α-GTase-negative); SD_282_: *L. fermentum* PFC282 (4,3-α-GTase-positive); SD_268_: *L. fermentum* PFC268 (4,3-α-GTase-negative). Subscripts “L” and “H” represent low (10^2^ CFU/g) and high (10^6^ CFU/g) initial inoculation levels, respectively.

Genus-level taxonomic profiles, obtained after filtering plant organelle sequences (chloroplast and mitochondria) and converting the data to relative abundances, suggested that LAB heavily dominated the microbiota structure. Across all analyzed samples, the genera *Lactobacillus*, *Weissella*, *Leuconostoc*, *Lactococcus*, and *Pediococcus* constituted the core components of the bacterial community (Figure 3).

**Figure 4:**
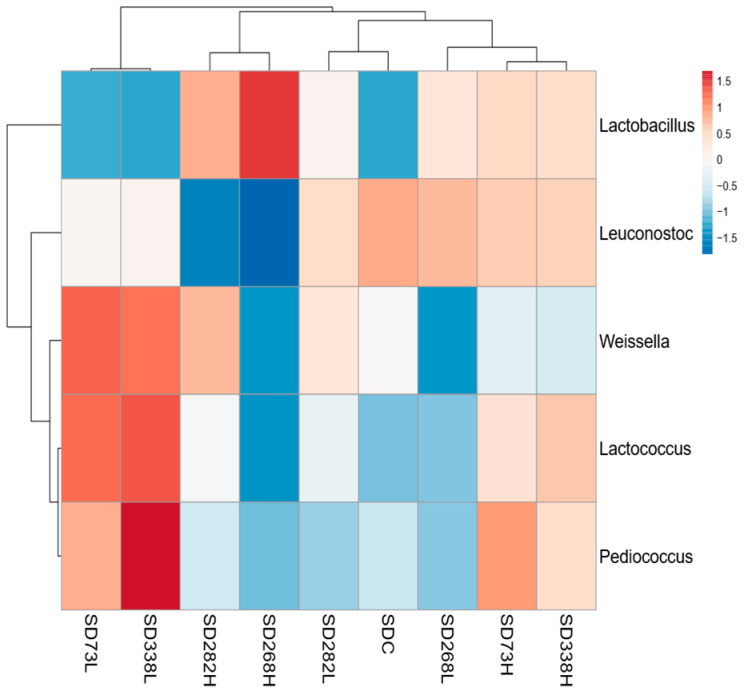
Heatmap and hierarchical clustering analysis showing the relative abundance and distribution of the most dominant bacterial genera across sourdough samples. Data were ln(x + 1) transformed and standardized using Z-scores. The color scale indicates relative intensity (red: higher abundance; blue: lower abundance compared to the mean). Rows represent bacterial genera, and columns represent sourdough groups. Samples are color-coded according to the starter strain and inoculation level (L: 10^2^ CFU/g; H: 10^6^ CFU/g). SD_C_: Spontaneous sourdough control; SD_338_: *L. reuteri* PFC338 (4,6-α-GTase-positive); SD_73_: *L. reuteri* PSC73 (4,6-α-GTase-negative); SD_282_: *L. fermentum* PFC282 (4,3-α-GTase-positive); SD_268_: *L. fermentum* PFC268 (4,3-α-GTase-negative).

Compared to SD_C_, starter culture inoculation induced distinct shifts in the bacterial composition. Notably, in the SD_268H_ sample, inoculated with a high dosage of the 4,3-α-GTase-negative *L. fermentum* PFC268 strain, the genus *Lactobacillus* constituted most of the population with a high relative abundance of 94.90%, leading to a lower relative abundance of other taxa. Conversely, the SD_338L_ sample, which featured the 4,6-α-GTase-positive *L. reuteri* PFC338 strain, maintained a co-existence of *Weissella* (40.90%), *Lactococcus* (22.98%), and *Lactobacillus* (18.83%) at high relative abundances. Similarly, in the SD_282L_ sample inoculated with the 4,3-α-GTase-positive *L. fermentum* PFC282 strain, *Lactobacillus* (39.83%) and *Leuconostoc* (23.02%) emerged as the dominant taxa, while the “others” group reached 23.85%, revealing a notable variation in taxonomic distribution (Figure 3). As a general trend, increasing the initial inoculation dosage (from 10^2^ to 10^6^ CFU/g) enhanced the dominance of the respective genera and reduced community heterogeneity. Hierarchical clustering and heatmap analysis further supported these dosage- and strain-associated similarity patterns among the samples (Figure 4).

**Figure 5:**
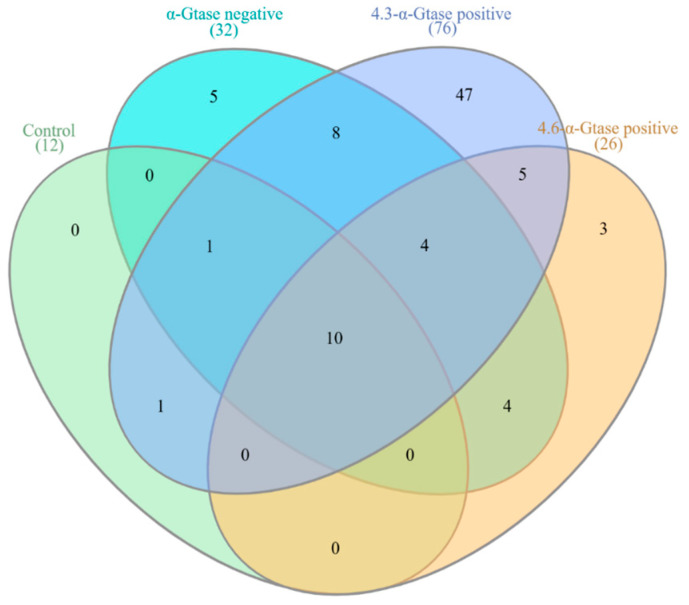
Venn diagram illustrating the shared and unique bacterial genera among the different sourdough groups. The four sets represent the spontaneous control (SD_C_), α-GTase-negative strains (*L. reuteri* PSC73 and *L. fermentum* PFC268), 4,3-α-GTase-positive strain (*L. fermentum* PFC282), and 4,6-α-GTase-positive strain (*L. reuteri* PFC338). Overlapping areas display the number of common genera, while non-overlapping areas indicate the unique taxonomic features of each group.

The hierarchical clustering tree indicated that the high-dose samples SD_73H_ and SD_338H_, which had similar compositions, clustered within the same primary group, while the taxonomically uniform SD_268H_ sample formed a distinct branch. A Venn diagram further delineated the shared and unique genera among the groups (Figure 5).

The Venn diagram revealed that 10 genera were shared among all analyzed sourdough groups (Figure 5). There were five specific genera present exclusively in both enzyme-positive groups (4,3- and 4,6-α-GTase) but completely absent from the spontaneous control and α-GTase-negative groups. Furthermore, the 4,3-α-GTase-positive group alone harbored 47 unique genera.

### 3.7. Beta Diversity and Community Structure

**Figure 6:**
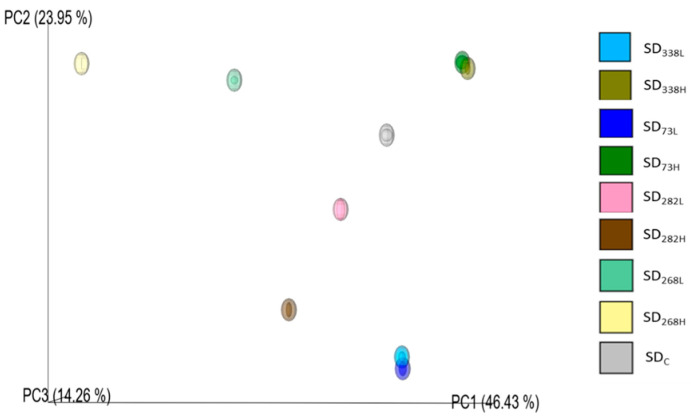
Principal Coordinates Analysis (PCoA) plot of bacterial communities based on distance matrices. Samples are color-coded according to the starter strain and inoculation level (L: 10^2^ CFU/g; H: 10^6^ CFU/g), with percentages on the axes indicating the proportion of total variation explained by each coordinate. SD_C_: Spontaneous sourdough control; SD_338_: *L*. *reuteri* PFC338 (4,6-α-GTase-positive); SD_73_: *L*. *reuteri* PSC73 (4,6-α-GTase-negative); SD_282_: *L*. *fermentum* PFC282 (4,3-α-GTase-positive); SD_268_: *L*. *fermentum* PFC268 (4,3-α-GTase-negative).

PCoA analysis based on distance matrices, performed to evaluate the bacterial community similarities among the samples, suggested that the starter culture type and inoculation level distinctly influenced the sourdough ecosystem (Figure 6).

The first two principal components in Figure 6 explained 70.38% of the total variation (PC1: 46.43%; PC2: 23.95%), delineating the taxonomic variation patterns among the groups. While SD_C_ occupied a central position on the PCoA plot, starter culture inoculation shifted the bacterial profiles in distinct directions. Notably, samples SD_268H_ (light yellow) and SD_268L_ (light green) separated from both SD_C_ and all other groups, positioning themselves on the far-left outer margins of the plot. Conversely, the samples inoculated with *L. reuteri* strains (PFC338 and PSC73) exhibited a taxonomic convergence. The high-dose samples SD_338H_ (olive green) and SD_73H_ (dark green) clustered closely together in the upper right quadrant, while the low-dose samples SD_338L_ (light blue) and SD_73L_ (dark blue) were tightly grouped in the lower right quadrant. In contrast, the samples inoculated with *L. fermentum* PFC282 strain (pink and brown) were widely separated.

### 3.8. Viscosity and Rheological Properties of Sourdoughs

The Power Law model evaluated the rheological flow behavior and viscosity parameters of the matured sourdough preferments prepared at an inoculation level of 10^6^ CFU/g at the end of the third backslopping cycle (BC_3_) for use as breadmaking starters (Table 4).

According to the rheological modeling data, the flow behavior index values of all samples, including SD_C_, were below 1. There was no statistically significant (*p* > 0.05) difference among the flow behavior index values of the samples. Conversely, starter culture inoculation generally induced a statistically significant (*p* < 0.05) variations in the consistency coefficient values. While the consistency coefficient of SD_C_ was 3.45 Pa.s*^n^*, all sourdoughs fermented with starter cultures, except for SD_268H_, exhibited significantly higher (*p* < 0.05) consistency coefficient values compared to SD_C_.

### 3.9. Specific Volume and Loaf Characteristics of Sourdough Breads

Table 5 summarizes the specific volume and loaf characteristics of the breads produced from the sourdough samples.

There was no statistically significant difference (*p* > 0.05) among the loaf weights (137.30–139.70 g) and baking loss rates (12.69–14.19%) of the sourdough bread samples. Conversely, starter culture inoculation exerted a significant effect (*p* < 0.05) on specific volume values. The SD_282H_-Bread sample, prepared with the 4,3-α-GTase-positive *L. fermentum* PFC282 strain, recorded the highest loaf volume (542.50 mL) and specific volume (3.88 mL/g) values (*p* < 0.05). Similarly, the SD_338H_-Bread sample produced with the 4,6-α-GTase-positive *L. reuteri* PFC338 strain led to a significant increase in loaf specific volume (3.61 mL/g) compared to SD_C_-Bread (3.34 mL/g) (*p* < 0.05). However, the specific volumes of the SD_268H_-Bread (3.47 mL/g) and SD_73H_-Bread (3.19 mL/g) samples, produced with α-GTase-negative strains, were statistically significantly lower (*p* < 0.05) than those of the breads produced with α-GTase-positive strains.

### 3.10. Textural Properties of Sourdough Breads During Storage

Table 6 summarizes the textural properties of the breads produced from the sourdough samples evaluated over a seven-day storage period.

All bread samples showed statistically significant (*p* < 0.05) increases in hardness values on the final day of storage (day 7) compared to the beginning of storage. On day 0, no statistically significant differences (*p* > 0.05) were observed in the hardness values among all bread samples, which ranged from 2.11 N to 3.72 N. By the final day of storage, while SD_C_-Bread and SD_73H_-Bread reached the highest hardness levels with values of 23.42 N and 24.24 N, respectively, the hardness of SD_282H_-Bread (11.53 N) remained significantly lower (*p* < 0.05) than all other samples.

Overall, the chewiness and gumminess values of the breads increased as the storage period extended; however, this increase varied among the samples and exhibited minor fluctuations. For the SD_282H_-Bread sample, chewiness values stabilized after day 3, remaining statistically similar (*p* > 0.05) among days 3, 5, and 7. Notably, on the final day of storage, SD_282H_-Bread distinguished itself by exhibiting a statistically significantly (*p* < 0.05) lower gumminess value compared to the other samples. Specifically, while SD_C_-Bread reached a gumminess of 7.36 N and a chewiness of 53.35 N, the gumminess and chewiness of SD_282H_-Bread were 4.76 N and 36.44 N, respectively (*p* < 0.05).

The cohesiveness decreased significantly (*p* < 0.05) in all samples as the storage period extended. Among the fresh breads, all breads produced using starter cultures possessed significantly higher (*p* < 0.05) cohesiveness values (0.78–0.80) compared to SD_C_-Bread (0.74). At the end of storage, the cohesiveness value of SD_C_-Bread dropped to 0.38, whereas the cohesiveness values of the SD_338H_-Bread and SD_268H_-Bread samples were 0.43 (*p* < 0.05). The springiness value of all samples, except for SD_268H_-Bread, decreased significantly (*p* < 0.05) on the final day compared to day 0 of storage. Conversely, the springiness value of SD_268H_-Bread remained similar (*p* > 0.05) throughout the storage period. Among all samples, SD_C_-Bread and SD_338H_-Bread exhibited the lowest springiness values on day 7.

### 3.11. Thermal Properties of Sourdough Breads During Storage

**Figure 7:**
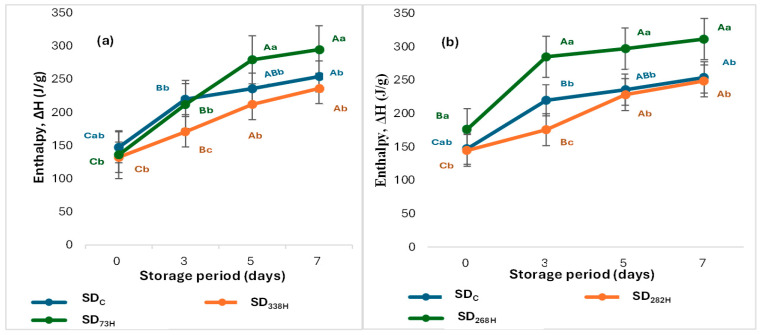
Changes in amylopectin retrogradation enthalpies (ΔH) of sourdough breads during seven days of storage: (**a**) 4,6-AGT group breads and (**b**) 4,3-AGT group breads. Error bars represent SD (n = 3). Two-way ANOVA revealed a significant sample × storage day interaction (*p* < 0.001). Different uppercase superscript letters within the same sample indicate statistically significant (*p* < 0.05) differences across storage days, and different lowercase superscript letters within the same storage day indicate statistically significant (*p* < 0.05) differences among bread samples. SD_C_-Bread: Spontaneous sourdough control; SD_338H_-Bread: *L. reuteri* PFC338 (10^6^ CFU/g); SD_73H_-Bread: *L. reuteri* PSC73 (10^6^ CFU/g); SD_282H_-Bread: *L. fermentum* PFC282 (10^6^ CFU/g); SD_268H_-Bread: *L. fermentum* PFC268 (10^6^ CFU/g).

Figure 7 summarizes the thermal properties of the breads over a seven-day storage period.

Throughout the storage period, the endothermic peak onset temperatures (T_0_) for the melting of amylopectin crystals in all bread samples remained at approximately 40 °C. Both storage time and sample variations demonstrated a significant effect (*p* < 0.001) on the enthalpy (ΔH) values of the breads. Prolonging the storage time increased the retrogradation enthalpy values of all breads, and the difference between the first day (day 0) and the final day (day 7) of storage was significant (*p* < 0.05). At the beginning of storage, SD_268H_-Bread (176.46 J/g) and SD_C_-Bread (147.36 J/g) exhibited the highest initial enthalpy values (*p* > 0.05) (Figure 7b), whereas the other samples had significantly (*p* < 0.05) lower enthalpy values than SD_268H_-Bread. On day 3 of storage, the SD_338H_-Bread (170.88 J/g) and SD_282H_-Bread (175.90 J/g) samples produced with α-GTase-positive strains showed the lowest enthalpies (*p* < 0.05) among all samples. Conversely, on the same day, SD_268H_-Bread (285.01 J/g), had the highest enthalpy (*p* < 0.05). On days 5 and 7, although significantly lower (*p* < 0.05) than the other samples, the enthalpy values of the SD_338H_-Bread, SD_282H_-Bread, and SD_C_-Bread samples remained similar (*p* > 0.05) to one another. At the end of storage, the sample with the lowest amylopectin retrogradation enthalpy peak was SD_338H_-Bread (236.10 J/g) (Figure 7a), while the samples with the highest retrogradation enthalpy values were SD_268H_-Bread (311.63 J/g) (Figure 7b) and SD_73H_-Bread (294.54 J/g), respectively. The enthalpy values of all samples remained similar (*p* > 0.05) between days 5 and 7.

### 3.12. Estimated Glycemic Index of Sourdough Breads

Table 7 presents the hydrolysis index (HI), estimated glycemic index (eGI), and glycemic category results of the bread samples.

Sourdough fermentation with different starter cultures demonstrated a significant reducing effect (*p* < 0.05) on the HI and eGI values of the breads compared to the reference white bread, which was classified in the “high-glycemic” category. The SD_338H_-Bread sample, produced with the 4,6-α-GTase-positive *L. reuteri* PFC338 strain, scored an HI of 52.99 and an eGI of 68.80; these values were significantly lower (*p* < 0.05) than those of all other samples, making it the only sample in the “medium-glycemic” category. Conversely, the remaining sourdough formulations (SD_73H_-Bread, SD_282H_-Bread and SD_268H_-Bread) still remained within the “high-glycemic” category, exhibiting statistically similar (*p* > 0.05) eGI values to one another.

## 4. Discussion

The selection of paired α-GTase-positive and -negative models within the same species (*L. reuteri* and *L. fermentum*) provided a framework for tracking how the starch matrix undergoes restructuring during fermentation. Both *L. reuteri* and *L. fermentum*, frequently isolated from sourdough ecosystems [28], possess documented capabilities to synthesize α-GTase enzymes [12,29].

The TTA results (0.48–0.72%) obtained during fermentation align with the lower limits reported by Syrokou et al. [30], who documented TTA values of 0.50–1.59% in wheat sourdoughs. The high TTA levels (0.71–0.72%) maintained by sample SD_338H_ throughout all BCs suggest that the *L. reuteri* PFC338 strain rapidly adapted to the dough environment, contributing to a stable establishment in the matrix. The stability of the LAB and TAMB loads between 8 and 9.30 log CFU/g across successive BCs supports the overall adaptability of the microflora under these fermentation conditions. This microbial stability and acidity development likely created a biopreservative barrier against other competitive microorganisms in the ecosystem. Indeed, the decline in yeast and mold counts as the cycles progressed could be attributed to the antifungal activity of organic acids or potential compounds like reuterin synthesized particularly by *L. reuteri* strains [31]. The high and stable metabolic activity of the LAB population suggests the successful establishment of the biotic infrastructure required for in situ α-GTase functionality in the dough rheology and starch matrix during the subsequent stages.

The DGGE profiles of the samples at BC_3_ indicated an impact of starter culture inoculation on the bacterial profiles. The addition of LAB starters led to a reduction in the intensity of the complex microbial band clusters present in SD_C_. This outcome suggests that the starter cultures adapted to the ecosystem, influencing the overall bacterial band profile. Indeed, the literature [32] indicates that target LAB species dominate at the end of sourdough fermentations initiated with a high inoculation dosage, although the DGGE band profile may still include other strains of the same species naturally present in the flour microflora. However, the presence of bands in distinct positions and variations in band intensities reflect potential shifts within the dough matrix.

The lower Chao1 value observed in SD_C_ can be attributed to the selective proliferation of specific dominant taxa from the native flour microflora that adapted to the fermentation conditions. Conversely, the overall increase in taxonomic richness following starter culture addition suggests that the starter culture inoculation enriched the sourdough matrix and expanded the existing microflora diversity. The variations observed in the Shannon and Simpson indices across different strains highlight the potential role of starter culture genotypic traits and inoculation dosages in modulating microbial profiles. Particularly when comparing the 4,3-α-GTase variants, the enzyme-active *L. fermentum* PFC282 strain at a low dosage (SD_282L_) promoted a more balanced and stratified community structure, yielding the highest Shannon index along with a distinct Simpson dominance profile within the dataset. In contrast, using the 4,3-α-GTase-negative *L. fermentum* PFC268 strain at a high inoculation level (SD_268H_) markedly reduced the evenness and diversity in the ecosystem compared to other samples. This 4,3-α-GTase-negative strain, inoculated at a high population (10^6^ CFU/g), free from the potential metabolic burden of *in situ* enzyme synthesis, and acid-tolerant, likely dominated the limited carbon sources in the dough environment, limiting the relative abundance of alternative taxa and leading to a community structure with low evenness [28,33]. The genus-level taxonomic distribution of the samples supports this trend. While the genus *Lactobacillus* was predominantly represented in SD_268H_, the samples inoculated with low dosages of α-GTase-positive strains (SD_338L_ and SD_282L_) maintained a more balanced and heterogeneous distribution of *Lactobacillus, Weissella, Lactococcus*, and *Leuconostoc.* This outcome can be tentatively attributed to the *in situ* α-GTase activity at low initial populations, which potentially generates alternative prebiotic carbon sources, such as modified glucans or oligomers (e.g., isomalto-oligosaccharides), within the dough matrix, thereby supporting bacterial homeostasis [33]. However, the increased *Lactobacillus* dominance and reduced heterogeneity in samples inoculated with high dosages of the same strains (SD_338H_ and SD_282H_) were likely driven by the intense metabolic competition imposed by the initial cell load. The Venn diagram results identified 10 shared genera forming the “core bacterial community” of the system, while the absence of unique taxa in the SD_C_ indicates that the starter cultures modified the bacterial profile of the dough. The presence of 47 unique genera in the samples produced with the 4,3-α-GTase-positive strain suggests that the diversity of *in situ* synthesized structural carbohydrates expanded through specific synergistic and metabolic interactions established between the starter culture and the flour microflora. Furthermore, the distribution of samples on the PCoA coordinate plot indicates that the bacterial community structure was shaped not only by the starter culture species but also by the strain genotype and inoculation dosage. The positioning of *L. fermentum* PFC268-fermented samples (SD_268L_ and SD_268H_) on the far-left outer margin of the coordinate axis, separated from the other samples, further supports the high adaptability and subsequent impact of this starter culture on the bacterial profile. Conversely, the distinct taxonomic convergence displayed by the doughs inoculated with both *L. reuteri* strains (PFC338 and PSC73), clustering according to inoculation dosages rather than enzymatic variations, suggests that the compositional stabilization capacity of *L. reuteri* in the flour matrix relates to its species-specific metabolic traits rather than the presence of α-GTase [28].

All samples exhibited a shear-thinning flow behavior, which is characteristic of flour-based matrices [34,35]. The statistically similar (*p* > 0.05) flow behavior index values among the samples indicate that starter culture variations and enzymatic activities did not alter the fundamental flow mechanism of the dough. Conversely, starter culture inoculation generally induced statistically significant (*p* < 0.05) variations in consistency coefficient values, with the SD_268H_ sample showing values statistically comparable (*p* > 0.05) to the SD_C_. The higher consistency coefficients in most of the starter-inoculated preferments compared to SD_C_ can be attributed to the exopolysaccharides (EPS) potentially synthesized by these strains during fermentation [36].

The improved specific volume values recorded for the breads produced with the 4,3-α-GTase-positive *L. fermentum* PFC282 (SD_282H_-Bread) and 4,6-α-GTase-positive *L. reuteri* PFC338 (SD_338H_-Bread) strains are consistent with the core hypothesis of this study. Notably, this volumetric performance exceeds the basal specific volume values (2.13 mL/g) reported by Verdonck et al. [37] for breads produced using sourdough. Furthermore, our findings align with a previous study [38] reporting a 2.3–11.4% increase in bread specific volume compared to the control upon adding the 4,6-α-GTase enzyme to the dough environment. The higher specific volumes of the α-GTase-positive samples compared to both SD_C_ and the breads produced with α-GTase-negative strains (Table 5) can be attributed to the *in situ* synthesis of modified α-glucans and oligosaccharides via enzymatic transglucosylation. Similar to the network-building and water-binding behavior of microbial hydrocolloids (such as dextrans) in cereal systems, these polysaccharide structures likely exerted a hydrocolloid-like effect within the dough matrix, interacting favorably with the gluten network to enhance dough stability and gas retention capacity [39]. In addition to this enzymatic mechanism, alternative and contributory biochemical factors, such as moderate acidification affecting the gluten network, microbial proteolysis influencing dough extensibility, and *in situ* EPS production [40], may have also affected bread specific volumes by modifying dough rheology [37]. Additionally, sugars like glucose and maltose released as a result of the enzymatic reaction may have provided an extra carbon source, thereby stimulating greater CO_2_ production [41].

The general increasing trend observed in the hardness, gumminess, and chewiness values of the breads during the seven-day storage period (Table 6), along with the decrease in cohesiveness and springiness values, can be attributed to the retrogradation of starch molecules gelatinized during baking and the migration of water from the crumb to the crust [3]. On day 0, no significant differences (*p* > 0.05) in hardness were observed across the freshly baked samples. However, when these textural profiles are evaluated together with thermal dynamics (Figure 7) it is shown that on the final day of storage, the SD_282H_-Bread sample exhibited significantly lower (*p* < 0.05) hardness and gumminess compared to all other samples, which aligns with the literature data reporting that α-GTase enzyme activity reduces crumb hardness, gumminess, and chewiness at the end of storage [38]. This anti-staling effect can be associated with the ability of the *L*. *fermentum* PFC282 strain to alter the branching architecture of flour starch through its *in situ* transferase activity [38] and to synthesize structural EPSs [42]. The general increase observed in the enthalpy values during bread storage demonstrates at the molecular level that starch chains recrystallize over time and enter the staling process [43]. Specifically, during the early stages of storage (day 3), breads produced with α-GTase-positive strains (SD_338H_-Bread and SD_282H_-Bread) exhibited significantly lower (*p* < 0.05) retrogradation enthalpies compared to breads produced with α-GTase-negative strains (SD_73H_-Bread and SD_268H_-Bread) and the SD_C_-Bread, suggesting a delayed crystallization rate. This thermal stability observed during the first 3 days of storage can be potentially explained by the ability of the α-GTase enzyme to disrupt the linear symmetry of amylopectin by increasing the number of short branches and the ratio of α-(1 → 6) linkages in the starch chains; this modification geometrically hinders the formation of regular crystal lattices and slows down the crystallization rate [38]. However, toward the end of the storage period (on days 5 and 7), the enthalpy values of the SD_338H_-Bread and SD_282H_-Bread samples were similar to that of SD_C_-Bread, implying that α-GTase enzyme activity slows down crystallization kinetics in the early storage phase rather than completely halting amylopectin retrogradation.

The decrease observed in the HI and eGI values of the breads compared to white bread can be attributed to the organic acids formed during sourdough fermentation, which limit starch gelatinization and promote gluten–starch interactions that reduce starch digestibility [44]. The observation that the SD_338H_-Bread sample, produced with *L. reuteri* PFC338, exhibited the lowest eGI value among all samples, becoming the only sample classified in the “medium-glycemic” category, can be linked to the ability of the 4,6-α-GTase enzyme to cleave α-(1 → 4) glycosidic bonds in starch molecules and transfer glucose residues to α-(1 → 6) positions. This enzymatic modification potentially increases the branching density of starch and establishes novel linkage structures unrecognized by digestive enzymes. Consequently, it reduces rapidly digestible starch fractions while promoting the formation of slowly digestible and resistant starches, thereby lowering the eGI by reducing the hydrolysis rate of amylolytic enzymes and delaying glucose release [38,45]. Conversely, the other α-GTase-positive sample, *L. fermentum* PFC282 (SD_282H_-Bread), remained in the “high-glycemic” group similarly to the α-GTase-negative strains (SD_73H_-Bread and SD_268H_-Bread) suggesting that enzyme specificity exerts molecular impacts on starch architecture. These findings highlight the potential of utilizing the in situ 4,6-α-GTase activity of *L. reuteri* PFC338 to develop functional bakery products with a reduced eGI.

Despite these observed physical and technological changes, a key limitation of this study is that the *in situ* expression or specific enzymatic activity of the 4,6-α- and 4,3-α-GTases during sourdough fermentation was not directly measured using enzyme assays or RT-qPCR of *gtfB* genes. The suggested structural modifications of starch and the subsequent changes were deduced from genetic confirmation and the observed differences between the α-GTase-positive and -negative strains. Therefore, future studies focusing on proteomic confirmation or starch linkage analysis are needed to clarify the expression kinetics of these enzymes within the sourdough matrix. Furthermore, since the glycemic properties in this study were evaluated solely through *in vitro* starch hydrolysis and human postprandial glycemic responses were not measured, future *in vivo* clinical trials are required to validate these potential metabolic benefits.

## 5. Conclusions

This study demonstrates that α-GTase-positive starter cultures were associated with improved bread quality and lower estimated starch digestibility in sourdough ecosystems. The selected *L. reuteri* and *L. fermentum* starter cultures successfully adapted to the matrix, modifying the native flour bacterial profile to establish stable acidity. Furthermore, high-throughput sequencing revealed that the inoculation level and strain genotype shaped the bacterial assembly, where lower-dose α-GTase-positive starters preserved a balanced taxonomic evenness while high-dose inoculation led to pronounced *Lactobacillus* prevalence. The use of these strains corresponded to enhanced apparent viscosity without altering characteristically pseudoplastic flow behavior, along with significantly improved bread specific volume. During storage, the application of the 4,3-α-GTase-positive *L. fermentum* PFC282 strain was linked to retarded amylopectin retrogradation, thereby significantly reducing hardness and gumminess, alongside lowering chewiness, to exert a distinct anti-staling effect. Crucially, the 4,6-α-GTase-positive *L. reuteri* PFC338 strain showed an association with lower enzymatic hydrolysis, which may be related to potential modifications in structural linkages, thereby emerging as a prominent strain profile capable of shifting bread from the “high-glycemic” to the “medium-glycemic” category. In conclusion, the application of α-GTase-positive starter cultures holds significant potential for the development of both long shelf life and reduced eGI clean-label products in the bakery industry, while further mechanistic investigations are warranted to fully elucidate in situ enzymatic pathways.

## Figures and Tables

**Table 1:** Experimental design applied for sourdough production carried out at 30 °C.

Starter Culture	Inoculation Rate (CFU/g)	Sample Codes
*L*. *reuteri* PFC338 (4,6-α-GTase-positive)	10^2^	SD_338L_
*L*. *reuteri* PFC338 (4,6-α-GTase-positive)	10^6^	SD_338H_
*L*. *reuteri* PSC73 (4,6-α-GTase-negative)	10^2^	SD_73L_
*L*. *reuteri* PSC73 (4,6-α-GTase-negative)	10^6^	SD_73H_
*L*. *fermentum* PFC282 (4,3-α-GTase-positive)	10^2^	SD_282L_
*L*. *fermentum* PFC282 (4,3-α-GTase-positive)	10^6^	SD_282H_
*L*. *fermentum* PFC268 (4,3-α-GTase-negative)	10^2^	SD_268L_
*L*. *fermentum* PFC268 (4,3-α-GTase-negative)	10^6^	SD_268H_
Control *	-	SD_C_

* No starter culture was added to the control sample.

**Table 2:** Total titratable acidity (TTA) and microbiological profiles of sourdoughs across successive backslopping cycles.

Sample	Backslopping Cycle	TTA	LAB * (on MRS)	LAB * (on GM17)	TAMB *	Yeast and Mold *
SD_C_	T_0_	0.60 ± 0.02 ^a^	8.47 ± 0.01 ^cd^	8.73 ± 0.04 ^bc^	8.58 ± 0.11 ^b^	3.67 ± 0.20 ^b^
	BC_1_	0.62 ± 0.02 ^abc^	8.80 ± 0.03 ^abc^	8.60 ± 0.01 ^b^	9.00 ± 0.10 ^a^	3.25 ± 0.08 ^ab^
	BC_2_	0.48 ± 0.01 ^de^	8.80 ± 0.03 ^ab^	8.60 ± 0.01 ^cd^	9.23 ± 0.23 ^a^	3.29 ± 0.01 ^c^
	BC_3_	0.48 ± 0.02 ^b^	8.83 ± 0.06 ^bc^	9.04 ± 0.05 ^a^	8.75 ± 0.15 ^abc^	3.32 ± 0.04 ^c^
SD_338L_	T_0_	0.59 ± 0.03 ^a^	8.45 ± 0.15 ^de^	9.15 ± 0.15 ^a^	8.45 ± 0.15 ^bc^	3.16 ± 0.09 ^c^
	BC_1_	0.49 ± 0.03 ^d^	8.60 ± 0.30 ^bcd^	8.73 ± 0.04 ^b^	9.00 ± 0.10 ^a^	3.29 ± 0.02 ^ab^
	BC_2_	0.49 ± 0.00 ^de^	9.23 ± 0.23 ^a^	9.15 ± 0.15 ^ab^	9.15 ± 0.15 ^a^	3.09 ± 0.05 ^d^
	BC_3_	0.48 ± 0.06 ^b^	9.15 ± 0.15 ^ab^	9.15 ± 0.15 ^a^	9.15 ± 0.15 ^a^	ND
SD_338H_	T_0_	0.68 ± 0.09 ^a^	8.00 ± 0.10 ^f^	8.10 ± 0.10 ^d^	8.00 ± 0.00 ^d^	3.46 ± 0.00 ^bc^
	BC_1_	0.72 ± 0.01 ^a^	8.45 ± 0.15 ^cd^	8.60 ± 0.00 ^b^	8.60 ± 0.10 ^ab^	ND
	BC_2_	0.72 ± 0.03 ^a^	9.23 ± 0.23 ^a^	9.23 ± 0.23 ^a^	9.00 ± 0.10 ^a^	ND
	BC_3_	0.71 ± 0.06 ^a^	9.30 ± 0.10 ^a^	9.30 ± 0.10 ^a^	8.60 ± 0.30 ^bc^	ND
SD_73L_	T_0_	0.70 ± 0.09 ^a^	9.15 ± 0.15 ^a^	8.83 ± 0.23 ^bc^	9.30 ± 0.10 ^a^	3.19 ± 0.05 ^c^
	BC_1_	0.60 ± 0.01 ^abcd^	9.00 ± 0.10 ^ab^	9.00 ± 0.10 ^a^	8.38 ± 0.38 ^b^	2.83 ± 0.06 ^c^
	BC_2_	0.53 ± 0.02 ^bcd^	9.15 ± 0.15 ^a^	9.15 ± 0.15 ^ab^	8.83 ± 0.06 ^a^	3.11 ± 0.00 ^d^
	BC_3_	0.69 ± 0.13 ^a^	8.83 ± 0.06 ^bc^	9.00 ± 0.10 ^a^	8.80 ± 0.20 ^abc^	ND
SD_73H_	T_0_	0.59 ± 0.02 ^a^	8.30 ± 0.00 ^def^	8.30 ± 0.00 ^d^	8.15 ± 0.15 ^cd^	3.62 ± 0.26 ^b^
	BC_1_	0.57 ± 0.10 ^bcd^	9.15 ± 0.15 ^a^	9.00 ± 0.00 ^a^	9.00 ± 0.00 ^a^	ND
	BC_2_	0.49 ± 0.02 ^de^	8.77 ± 0.30 ^ab^	8.72 ± 0.12 ^c^	9.15 ± 0.15 ^a^	ND
	BC_3_	0.53 ± 0.05 ^ab^	8.83 ± 0.06 ^bc^	9.00 ± 0.10 ^a^	8.77 ± 0.17 ^abc^	ND
SD_282L_	T_0_	0.59 ± 0.03 ^a^	8.15 ± 0.15 ^ef^	8.64 ± 0.01 ^c^	9.00 ± 0.10 ^a^	4.54 ± 0.02 ^a^
	BC_1_	0.54 ± 0.05 ^bcd^	8.29 ± 0.01 ^cd^	9.00 ± 0.10 ^a^	8.15 ± 0.15 ^b^	3.32 ± 0.05 ^a^
	BC_2_	0.59 ± 0.04 ^b^	9.25 ± 0.05 ^a^	9.00 ± 0.10 ^abc^	9.00 ± 0.10 ^a^	3.07 ± 0.00 ^d^
	BC_3_	0.63 ± 0.08 ^ab^	8.64 ± 0.04 ^c^	9.00 ± 0.10 ^a^	9.00 ± 0.20 ^ab^	ND
SD_282H_	T_0_	0.40 ± 0.03 ^b^	8.76 ± 0.07 ^bc^	8.90 ± 0.10 ^abc^	9.25 ± 0.05 ^a^	ND
	BC_1_	0.64 ± 0.02 ^ab^	8.23 ± 0.23 ^d^	8.29 ± 0.01 ^c^	8.29 ± 0.01 ^b^	ND
	BC_2_	0.57 ± 0.01 ^bc^	8.38 ± 0.08 ^b^	8.80 ± 0.03 ^bc^	8.23 ± 0.23 ^b^	4.04 ± 0.02 ^a^
	BC_3_	0.56 ± 0.04 ^ab^	9.00 ± 0.10 ^ab^	8.53 ± 0.23 ^b^	9.10 ± 0.10 ^a^	4.01 ± 0.05 ^a^
SD_268L_	T_0_	0.58 ± 0.04 ^a^	9.00 ± 0.10 ^ab^	9.20 ± 0.05 ^a^	8.69 ± 0.00 ^b^	4.48 ± 0.00 ^a^
	BC_1_	0.51 ± 0.02 ^cd^	8.57 ± 0.27 ^bcd^	9.00 ± 0.10 ^a^	8.86 ± 0.09 ^a^	3.18 ± 0.04 ^b^
	BC_2_	0.50 ± 0.04 ^cde^	9.15 ± 0.15 ^a^	9.15 ± 0.15 ^ab^	8.92 ± 0.08 ^a^	3.13 ± 0.06 ^d^
	BC_3_	0.61 ± 0.05 ^ab^	9.20 ± 0.20 ^a^	9.30 ± 0.10 ^a^	8.79 ± 0.10 ^abc^	ND
SD_268 H_	T_0_	0.66 ± 0.01 ^a^	9.00 ± 0.10 ^ab^	9.00 ± 0.10 ^ab^	9.15 ± 0.15 ^a^	ND
	BC_1_	0.52 ± 0.04 ^bcd^	8.58 ± 0.11 ^bcd^	8.15 ± 0.15 ^c^	8.23 ± 0.23 ^b^	ND
	BC_2_	0.43 ± 0.02 ^e^	8.62 ± 0.15 ^b^	8.23 ± 0.23 ^d^	8.23 ± 0.23 ^b^	3.50 ± 0.04 ^b^
	BC_3_	0.54 ± 0.04 ^ab^	9.15 ± 0.15 ^ab^	8.54 ± 0.24 ^b^	8.38 ± 0.08	3.84 ± 0.05 ^b^

* Microbial populations are expressed in log CFU/g. Results represent mean values (n = 3) ± SD. Different lowercase superscript letters within the same column indicate statistically significant (*p* < 0.05) differences between sourdough groups within the same backslopping cycle (BC). T_0_ represents the unfermented fresh mix immediately after inoculation and prior to fermentation. TTA values are expressed as percentage (%) of lactic acid equivalents. LAB: Lactic acid bacteria; TAMB: Total aerobic mesophilic bacteria; SD_C_: Spontaneous sourdough control; SD_338_: *L. reuteri* PFC338 (4,6-α-GTase-positive); SD_73_: *L. reuteri* PSC73 (4,6-α-GTase-negative); SD_282_: *L. fermentum* PFC282 (4,3-α-GTase-positive); SD_268_: *L. fermentum* PFC268 (4,3-α-GTase-negative). Subscripts “L” and “H” represent low (10^2^ CFU/g) and high (10^6^ CFU/g) initial inoculation levels, respectively. ND: Not detected (below the detection limit of 1.00 log CFU/g).

**Table 3:** Alpha diversity metrics of composite pooled sourdough samples.

Sample	Chao1	Shannon	Simpson	Good’s Coverage
SD_C_	42.62 ± 5.85	3.24 ± 0.05	0.1814 ± 0.0101	0.9961 ± 0.0009
SD_338L_	75.64 ± 22.86	3.24 ± 0.03	0.1205 ± 0.0088	0.9912 ± 0.0024
SD_338H_	58.65 ± 10.09	3.24 ± 0.04	0.1077 ± 0.0067	0.9940 ± 0.0017
SD_73L_	62.79 ± 8.93	3.18 ± 0.05	0.1159 ± 0.0047	0.9926 ± 0.0014
SD_73H_	113.28 ± 25.78	3.09 ± 0.05	0.0656 ± 0.0039	0.9848 ± 0.0033
SD_282L_	54.57 ± 10.32	3.81 ± 0.02	0.2575 ± 0.0255	0.9945 ± 0.0016
SD_282H_	113.90 ± 35.14	3.40 ± 0.05	0.1386 ± 0.0080	0.9868 ± 0.0015
SD_268L_	52.58 ± 11.01	3.17 ± 0.03	0.1538 ± 0.0094	0.9944 ± 0.0016
SD_268H_	90.73 ± 16.44	2.08 ± 0.04	0.0584 ± 0.0041	0.9877 ± 0.0016

Data represents the mean values ± SD calculated across 10 computational rarefaction iterations performed at a standardized sequencing depth of 2000 reads per sample. SD_C_: Spontaneous sourdough control; SD_338_: *L. reuteri* PFC338 (4,6-α-GTase-positive); SD_73_: *L. reuteri* PSC73 (4,6-α-GTase-negative); SD_282_: *L. fermentum* PFC282 (4,3-α-GTase-positive); SD_268_: *L. fermentum* PFC268 (4,3-α-GTase-negative). Subscripts “L” and “H” represent low (10^2^ CFU/g) and high (10^6^ CFU/g) initial inoculation levels, respectively.

**Table 4:** Rheological parameters of sourdough preferments.

Sample	Consistency Coefficient (Pa.s*^n^*)	Flow Behavior Index (*n*)	R^2^
SD_C_	3.45 ± 1.08 ^b^	0.78 ± 0.11	0.98
SD_338H_	20.48 ± 6.54 ^a^	0.65 ± 0.04	0.99
SD_73H_	19.44 ± 6.19 ^a^	0.69 ± 0.05	0.99
SD_282H_	18.40 ± 4.22 ^a^	0.70 ± 0.04	0.98
SD_268H_	13.33 ± 2.92 ^ab^	0.76 ± 0.04	0.99

Results were expressed as mean values (n = 3) ± SD. Different lowercase superscript letters within the same column indicate statistically significant (*p* < 0.05) differences between sourdough samples. SD_C_: Spontaneous sourdough control; SD_338H_: *L*. *reuteri* PFC338 (10^6^ CFU/g); SD_73H_: *L. reuteri* PSC73 (10^6^ CFU/g); SD_282H_: *L. fermentum* PFC282 (10^6^ CFU/g); SD_268H_: *L. fermentum* PFC268 (10^6^ CFU/g).

**Table 5:** Technological and physical properties of sourdough breads.

Bread Sample	Loaf Weight (g)	Loaf Volume (mL)	Specific Volume (mL/g)	Baking Loss (%)
SD_C_-Bread	137.30 ± 1.35	458.00 ± 12.00 ^c^	3.34 ± 0.05 ^d^	14.19 ± 0.84
SD_338H_-Bread	138.65 ± 0.48	500.00 ± 5.77 ^b^	3.61 ± 0.04 ^b^	13.34 ± 0.30
SD_73H_-Bread	139.52 ± 1.22	455.00 ± 5.00 ^c^	3.19 ± 0.01 ^e^	12.80 ± 0.76
SD_282H_-Bread	139.70 ± 0.40	542.50 ± 12.50 ^a^	3.88 ± 0.08 ^a^	12.69 ± 0.25
SD_268H_-Bread	137.30 ± 2.60	476.00 ± 6.00 ^c^	3.47 ± 0.02 ^c^	14.19 ± 1.63

Results were expressed as mean values (n = 3) ± SD. Different lowercase superscript letters within the same column indicate statistically significant (*p* < 0.05) differences between sourdough samples. SD_C_-Bread: Spontaneous sourdough bread control; SD_338H_-Bread: *L. reuteri* PFC338 (10^6^ CFU/g); SD_73H_-Bread: *L. reuteri* PSC73 (10^6^ CFU/g); SD_282H_-Bread: *L. fermentum* PFC282 (10^6^ CFU/g); SD_268H_-Bread: *L. fermentum* PFC268 (10^6^ CFU/g).

**Table 6:** Textural profile analysis of sourdough breads during seven days of storage.

Textural Parameter	Bread Sample	Day 0	Day 3	Day 5	Day 7
Hardness (N)	SD_C_-Bread	3.72 ± 0.29 ^Da^	9.53 ± 0.69 ^Ca^	11.89 ± 0.71 ^Bb^	23.42 ± 1.64 ^Aa^
	SD_338H_-Bread	3.70 ± 0.10 ^Ca^	8.59 ± 0.88 ^Bab^	14.73 ± 0.78 ^Aa^	15.37 ± 1.00 ^Ab^
	SD_73H_-Bread	3.29 ± 0.47 ^Da^	7.18 ± 0.26 ^Cbc^	16.43 ± 0.98 ^Ba^	24.24 ± 1.71 ^Aa^
	SD_282H_-Bread	2.63 ± 0.14 ^Ca^	7.33 ± 0.21 ^Bbc^	9.03 ± 0.29 ^Bc^	11.53 ± 0.85 ^Ac^
	SD_268H_-Bread	2.11 ± 0.10 ^Da^	6.36 ± 0.28 ^Cc^	9.01 ± 0.37 ^Bc^	15.53 ± 0.99 ^Ab^
Chewiness (N)	SD_C_-Bread	22.04 ± 1.39 ^Cab^	39.63 ± 2.12 ^Ba^	50.57 ± 2.45 ^Aa^	53.35 ± 2.35 ^Aa^
	SD_338H_-Bread	22.57 ± 0.62 ^Ca^	37.87 ± 3.15 ^Ba^	52.88 ± 2.40 ^Aa^	52.05 ± 2.12 ^Aa^
	SD_73H_-Bread	23.24 ± 2.82 ^Ca^	39.07 ± 2.76 ^Ba^	51.61 ± 1.77 ^Aa^	51.70 ± 4.28 ^Aa^
	SD_282H_-Bread	16.87 ± 0.80 ^Bbc^	37.75 ± 0.70 ^Aa^	34.03 ± 1.43 ^Ab^	36.44 ± 3.26 ^Ab^
	SD_268H_-Bread	14.17 ± 0.41 ^Cc^	30.31 ± 1.04 ^Bb^	37.29 ± 1.42 ^Ab^	41.60 ± 1.58 ^Ab^
Cohesiveness	SD_C_-Bread	0.74 ± 0.01 ^Ab^	0.54 ± 0.01 ^Bb^	0.47 ± 0.01 ^Ca^	0.38 ± 0.01 ^Db^
	SD_338H_-Bread	0.78 ± 0.00 ^Aa^	0.57 ± 0.01 ^Bb^	0.50 ± 0.01 ^Ca^	0.43 ± 0.01 ^Da^
	SD_73H_-Bread	0.79 ± 0.01 ^Aa^	0.55 ± 0.02 ^Bb^	0.50 ± 0.02 ^Ca^	0.40 ± 0.01 ^Dab^
	SD_282H_-Bread	0.79 ± 0.01 ^Aa^	0.57 ± 0.01 ^Bb^	0.48 ± 0.02 ^Ca^	0.41 ± 0.01 ^Dab^
	SD_268H_-Bread	0.80 ± 0.01 ^Aa^	0.62 ± 0.02 ^Ba^	0.49 ± 0.01 ^Ca^	0.43 ± 0.02 ^Da^
Gumminess (N)	SD_C_-Bread	2.78 ± 0.18 ^Ca^	5.35 ± 0.48 ^Ba^	6.49 ± 0.44 ^Aa^	7.36 ± 0.35 ^Aab^
	SD_338H_-Bread	2.86 ± 0.07 ^Ca^	5.24 ± 0.45 ^Ba^	7.00 ± 0.41 ^Aa^	7.37 ± 0.44 ^Aab^
	SD_73H_-Bread	2.55 ± 0.32 ^Cab^	4.90 ± 0.40 ^Bab^	6.43 ± 0.38 ^Aa^	6.63 ± 0.66 ^Ab^
	SD_282H_-Bread	2.07 ± 0.09 ^Bab^	4.07 ± 0.08 ^Ab^	4.45 ± 0.24 ^Ab^	4.76 ± 0.41 ^Ac^
	SD_268H_-Bread	1.68 ± 0.08 ^Cb^	4.08 ± 0.24 ^Bb^	4.50 ± 0.20 ^Bb^	7.58 ± 0.80 ^Aa^
Springiness	SD_C_-Bread	7.95 ± 0.08 ^Aab^	7.71 ± 0.07 ^Bb^	7.70 ± 0.12 ^Bc^	7.39 ± 0.10 ^Cc^
	SD_338H_-Bread	7.87 ± 0.06 ^Ab^	7.73 ± 0.08 ^Ab^	7.69 ± 0.07 ^ABc^	7.54 ± 0.09 ^Bbc^
	SD_73H_-Bread	8.13 ± 0.07 ^Aa^	7.94 ± 0.13 ^Ba^	7.74 ± 0.13 ^Cbc^	7.72 ± 0.10 ^Cb^
	SD_282H_-Bread	8.14 ± 0.06 ^Aa^	8.05 ± 0.05 ^ABa^	7.93 ± 0.04 ^Bab^	7.65 ± 0.06 ^Cb^
	SD_268H_-Bread	8.12 ± 0.07 ^Aa^	8.01 ± 0.04 ^Aa^	8.01 ± 0.07 ^Aa^	7.98 ± 0.05 ^Aa^
ANOVA (*p* values)	Hardness (N)	Chewiness (N)	Cohesiveness	Gumminess (N)	Springiness
Sample (S)	*p* < 0.001	*p* < 0.001	*p* < 0.001	*p* < 0.001	*p* < 0.001
Day (D)	*p* < 0.001	*p* < 0.001	*p* < 0.001	*p* < 0.001	*p* < 0.001
Interaction (S × D)	*p* < 0.001	*p* < 0.001	*p* = 0.001	*p* < 0.001	*p* = 0.002

Results were expressed as mean values (n = 3) ± SD. Different lowercase superscript letters within the same column indicate statistically significant (*p* < 0.05) differences between bread samples for the same storage day. Different uppercase superscript letters within the same row indicate statistically significant (*p* < 0.05) differences between storage days (0, 3, 5, and 7 days) for the same sample. SD_C_-Bread: Spontaneous sourdough bread control; SD_338H_-Bread: *L. reuteri* PFC338 (10^6^ CFU/g); SD_73H_-Bread: *L. reuteri* PSC73 (10^6^ CFU/g); SD_282H_-Bread: *L. fermentum* PFC282 (10^6^ CFU/g); SD_268H_-Bread: *L. fermentum* PFC268 (10^6^ CFU/g).

**Table 7:** *In vitro* starch hydrolysis index, estimated glycemic index, and glycemic category of sourdough breads.

Bread Sample	Hydrolysis Index (HI)	Estimated Glycemic Index (eGI)	Glycemic Category
White Bread	100.00 ± 5.95 ^a^	94.61 ± 3.27 ^a^	High
SD_338H_-Bread	52.99 ± 0.63 ^c^	68.80 ± 0.35 ^c^	Medium
SD_73H_-Bread	83.31 ± 2.65 ^b^	85.45 ± 1.46 ^b^	High
SD_282H_-Bread	85.89 ± 1.98 ^b^	86.86 ± 1.09 ^b^	High
SD_268H_-Bread	91.41 ± 0.33 ^b^	89.90 ± 0.19 ^b^	High

Results were expressed as mean values (n = 3) ± SD. Different lowercase superscript letters within the same column indicate statistically significant (*p* < 0.05) differences between bread samples. SD_338H_-Bread: *L. reuteri* PFC338 (10^6^ CFU/g); SD_73H_-Bread: *L. reuteri* PSC73 (10^6^ CFU/g); SD_282H_-Bread: *L. fermentum* PFC282 (10^6^ CFU/g); SD_268H_-Bread: *L. fermentum* PFC268 (10^6^ CFU/g). Glycemic categories were classified based on Venn and Green [27] guidelines: low (<55), medium (55–69), and high (≥70).

## Data Availability

The data supporting the findings of this study are available within the article and its Supplementary Materials. The raw 16S rRNA amplicon sequencing datasets generated during this study are part of an ongoing research project. Due to ongoing project processes, the raw sequencing data are not publicly shared in a repository but can be made available from the corresponding author upon reasonable academic request.

## Supplementary Materials

The following supporting information can be downloaded at: https://www.mdpi.com/article/10.3390/foods15152757/s1, Figure S1: Rarefaction curves of sourdough samples based on observed features.

## References

[1] De Vuyst L., Comasio A., Kerrebroeck S.V. (2023). Sourdough production: Fermentation strategies, microbial ecology, and use of non-flour ingredients. Crit. Rev. Food Sci. Nutr..

[2] Mihhalevski A., Heinmaa I., Traksmaa R., Pehk T., Mere A., Paalme T. (2012). Structural changes of starch during baking and staling of rye bread. J. Agric. Food Chem..

[3] Fadda C., Sanguinetti A.M., Del Caro A., Collar C., Piga A. (2014). Bread staling: Updating the view. Compr. Rev. Food Sci. Food. Saf..

[4] Demirkesen-Bicak H., Arici M., Yaman M., Karasu S., Sagdic O. (2021). Effect of different fermentation condition on estimated glycemic index, in vitro starch digestibility, and textural and sensory properties of sourdough bread. Foods.

[5] Adewale P., Yancheshmeh M.S., Lam E. (2022). Starch modification for non-food, industrial applications: Market intelligence and critical review. Carbohydr. Polym..

[6] Bangar S.P., Ashogbon A.O., Singh A., Chaudhary V., Whiteside W.S. (2022). Enzymatic modification of starch: A green approach for starch applications. Carbohydr. Polym..

[7] BeMiller J.N., Huber K.C. (2015). Physical modification of food starch functionalities. Annu. Rev. Food Sci. Technol..

[8] Yang Y., Zhao X., Zhang T., Hamaker B.R., Miao M. (2021). Development of a novel starch-based dietary fiber using glucanotransferase. Food Funct..

[9] Miao M., BeMiller J.N. (2023). Enzymatic approaches for structuring starch to improve functionality. Annu. Rev. Food Sci. Technol..

[10] Chen Y., McClements D.J., Peng X., Chen L., Xu Z., Meng M., Ji H., Long J., Qiu C., Zhao J. (2023). Research progresses on enzymatic modification of starch with 4-α-glucanotransferase. Trends Food Sci. Technol..

[11] Kralj S., Grijpstra P., van Leeuwen S.S., Leemhuis H., Dobruchowska J.M., van der Kaaij R.M., Malik A., Oetari A., Kamerling J.P., Dijkhuizen L. (2011). 4,6-α-Glucanotransferase, a novel enzyme that structurally and functionally provides an evolutionary link between glycoside hydrolase enzyme families 13 and 70. Appl. Environ. Microbiol..

[12] Gangoiti J., van Leeuwen S.S., Gerwig G.J., Duboux S., Vafiadi C., Pijning T., Dijkhuizen L. (2017). 4,3-α-Glucanotransferase, a novel reaction specificity in glycoside hydrolase family 70 and clan GH-H. Sci. Rep..

[13] Li X., Fei T., Wang Y., Zhao Y., Pan Y., Li D. (2018). Wheat starch with low retrogradation properties produced by modification of the GtfB enzyme 4, 6-α-glucanotransferase from *Streptococcus thermophilus*. J. Agric. Food Chem..

[14] Leemhuis H., Dobruchowska J.M., Ebbelaar M., Faber F., Buwalda P.L., van der Maarel M.J.E.C., Kamerling J.P., Dijkhuizen L. (2014). Isomalto/malto-polysaccharide, a novel soluble dietary fiber made via enzymatic conversion of starch. J. Agric. Food Chem..

[15] Yang W., Sheng L., Chen S., Wang L., Su L., Wu J. (2022). Characterization of a new 4, 6-α-glucanotransferase from *Limosilactobacillus fermentum* NCC 3057 with ability of synthesizing low molecular mass isomalto-/maltopolysaccharide. Food Biosci..

[16] Bıyıklı A., Niçin R.T., Dertli E., Şimşek Ö. (2023). Extracellular recombinant production of 4,6 and 4,3 α-glucanotransferases in *Lactococcus lactis*. Enzym. Microb. Technol..

[17] Niçin R.T., Zehir-Şentürk D., Özkan B., Göksungur Y., Şimşek Ö. (2024). Optimization of 4,6-α and 4,3-α-Glucanotransferase production in *Lactococcus lactis* and determination of their effects on some quality characteristics of bakery products. Foods.

[18] Van der Meulen R., Scheirlinck I., Van Schoor A., Huys G., Vancanneyt M., Vandamme P., De Vuyst L. (2007). Population dynamics and metabolite target analysis of lactic acid bacteria during laboratory fermentations of wheat and spelt sourdoughs. Appl. Environ. Microbiol..

[19] Oguntoyinbo F.A., Dodd C.E. (2010). Bacterial dynamics during the spontaneous fermentation of cassava dough in gari production. Food Control.

[20] Bolyen E., Rideout J.R., Dillon M.R., Bokulich N.A., Abnet C.C., Al-Ghalith G.A., Alexander H., Alm E.J., Arumugam M., Asnicar F. (2019). Reproducible, interactive, scalable and extensible microbiome data science using QIIME 2. Nat. Biotechnol..

[21] Kazakos S., Mantzourani I., Plessas S. (2022). Quality characteristics of novel sourdough breads made with functional *Lacticaseibacillus paracasei* SP5 and prebiotic food matrices. Foods.

[22] Sun X., Ma L., Zhong X., Liang J. (2022). Potential of raw and fermented maize gluten feed in bread making: Assess of dough rheological properties and bread quality. LWT.

[23] Hadaegh H., Seyyedain Ardabili S.M., Tajabadi Ebrahimi M., Chamani M., Azizi Nezhad R. (2017). The impact of different lactic acid bacteria sourdoughs on the quality characteristics of toast bread. J. Food Qual..

[24] Englyst H.N., Kingman S.M., Cummings J.H. (1992). Classification and measurement of nutritionally important starch fractions. Eur. J. Clin. Nutr..

[25] Yaman M., Sargın H.S., Mızrak Ö.F. (2019). Free sugar content, in vitro starch digestibility and predicted glycemic index of ready-to-eat breakfast cereals commonly consumed in Turkey: An evaluation of nutritional quality. Int. J. Biol. Macromol..

[26] Goñi I., Garcia-Diz L., Mañas E., Saura-Calixto F. (1996). Analysis of resistant starch: A method for foods and food products. Food Chem..

[27] Venn B.J., Green T.J. (2007). Glycemic index and glycemic load: Measurement issues and their effect on diet–disease relationships. Eur. J. Clin. Nutr..

[28] De Vuyst L., Vrancken G., Ravyts F., Rimaux T., Weckx S. (2009). Biodiversity, ecological determinants, and metabolic exploitation of sourdough microbiota. Food Microbiol..

[29] Chen Y., Dong J., Li X., Jin Z., Svensson B., Bai Y. (2024). Acceptor subsite mutants of *Limosilactobacillus fermentum* NCC 2970 GtfB 4, 3-α-Glucanotransferase regulate the ratio of (α1→3)/(α1→6) linkages in biosynthesized α-Glucans. J. Agric. Food Chem..

[30] Syrokou M.K., Themeli C., Paramithiotis S., Mataragas M., Bosnea L., Argyri A.A., Chorianopoulos N.G., Skandamis P.N., Drosinos E.H. (2020). Microbial ecology of Greek wheat sourdoughs, identified by a culture-dependent and a culture-independent approach. Foods.

[31] Vimont A., Fernandez B., Ahmed G., Fortin H.P., Fliss I. (2019). Quantitative antifungal activity of reuterin against food isolates of yeasts and moulds and its potential application in yogurt. Int. J. Food Microbiol..

[32] Ravyts F., De Vuyst L. (2011). Prevalence and impact of single-strain starter cultures of lactic acid bacteria on metabolite formation in sourdough. Food Microbiol..

[33] Gänzle M.G. (2014). Enzymatic and bacterial conversions during sourdough fermentation. Food Microbiol..

[34] Hadnađev T.D., Pajić-Lijaković I., Hadnađev M., Mastilović J., Torbica A., Bugarski B. (2013). Influence of starch sodium octenyl succinate on rheological behaviour of wheat flour dough systems. Food Hydrocoll..

[35] Yazar G. (2023). Wheat flour quality assessment by fundamental non-linear rheological methods: A critical review. Foods.

[36] Coda R., Xu Y., Moreno D.S., Mojzita D., Nionelli L., Rizzello C.G., Katina K. (2018). Performance of *Leuconostoc citreum* FDR241 during wheat flour sourdough type I propagation and transcriptional analysis of exopolysaccharides biosynthesis genes. Food Microbiol..

[37] Verdonck C., De Bondt Y., Pradal I., Bautil A., Langenaeken N.A., Brijs K., Goos P., De Vuyst L., Courtin C.M. (2023). Impact of process parameters on the specific volume of wholemeal wheat bread made using sourdough-and baker’s yeast-based leavening strategies. Int. J. Food Microbiol..

[38] Li D., Zhao Y., Fei T., Wang Y., Lee B.H., Shim J.H., Xu B., Li Z., Li X. (2019). Effects of *Streptococcus thermophilus* GtfB enzyme on dough rheology, bread quality and starch digestibility. Food Hydrocoll..

[39] Lacaze G., Wick M., Cappelle S. (2007). Emerging fermentation technologies: Development of novel sourdoughs. Food Microbiol..

[40] Di Cagno R., De Angelis M., Limitone A., Minervini F., Carnevali P., Corsetti A., Gaenzle M., Ciati R., Gobbetti M. (2006). Glucan and fructan production by sourdough *Weissella cibaria* and *Lactobacillus plantarum*. J. Agric. Food Chem..

[41] Shim J.H., Seo N.S., Roh S.A., Kim J.W., Cha H., Park K.H. (2007). Improved bread-baking process using *Saccharomyces cerevisiae* displayed with engineered cyclodextrin glucanotransferase. J. Agric. Food Chem..

[42] Tieking M., Korakli M., Ehrmann M.A., Gänzle M.G., Vogel R.F. (2003). In situ production of exopolysaccharides during sourdough fermentation by cereal and intestinal isolates of lactic acid bacteria. Appl. Environ. Microbiol..

[43] Li C., Hu Y., Li E. (2021). Effects of amylose and amylopectin chain-length distribution on the kinetics of long-term rice starch retrogradation. Food Hydrocoll..

[44] Östman E.M., Nilsson M., Elmståhl H.L., Molin G., Björck I.M.E. (2002). On the effect of lactic acid on blood glucose and insulin responses to cereal products: Mechanistic studies in healthy subjects and in vitro. J. Cereal Sci..

[45] Ao Z., Simsek S., Zhang G., Venkatachalam M., Reuhs B.L., Hamaker B.R. (2007). Starch with a slow digestion property produced by altering its chain length, branch density, and crystalline structure. J. Agric. Food Chem..

