# Supplementary material for: In Situ Effects of 4,6-α- and 4,3-α-Glucanotransferases During Sourdough Fermentation: Assessing Microbial Community Dynamics, Bread Glycemic Index, Staling, and Texture
Source: Foods. 2026 Aug 5;15(15):2757. doi: 10.3390/foods15152757 (PMC13464822; doi:10.3390/foods15152757)
Supplement: Supplementary file 1 [file foods-15-02757-s001.zip › foods-4416763-supplementary.pdf]

**Supplementary Materials**

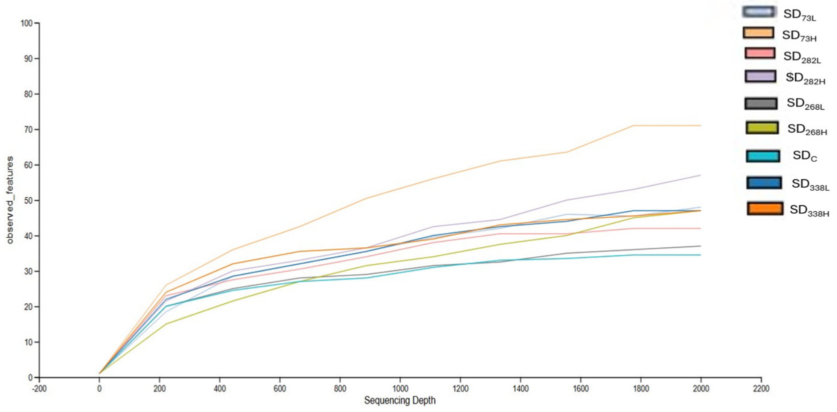


**Figure S1.** Rarefaction curves of sourdough samples based on observed features. The plateauing of the curves at a sequencing depth of 2000 reads indicates sufficient sampling of the microbial communities across all groups. SD$_{C}$: Spontaneous sourdough control; SD$_{338}$: *L. reuteri* PFC338 (4,6-α-GTase positive); SD$_{73}$: *L. reuteri* PSC73 (4,6-α-GTase negative); SD$_{282}$: *L. fermentum* PFC282 (4,3-α-GTase positive); SD$_{268}$: *L. fermentum* PFC268 (4,3-α-GTase negative). Subscripts "L" and "H" represent low ($10^2$ CFU/g) and high ($10^6$ CFU/g) initial inoculation levels, respectively.
